# Lifetime and Twelve-Month Prevalence, Persistence, and Unmet Treatment Needs of Mood, Anxiety, and Substance Use Disorders in African American and U.S. versus Foreign-Born Caribbean Women

**DOI:** 10.3390/ijerph17197007

**Published:** 2020-09-25

**Authors:** Audrey L. Jones, Susan D. Cochran, Jane Rafferty, Robert Joseph Taylor, Vickie M. Mays

**Affiliations:** 1 Informatics, Decision-Enhancement and Analytic Sciences Center (IDEAS), Veteran Affairs Salt Lake City Health Care System, Salt Lake City, UT 84148, USA; 2 Department of Internal Medicine, School of Medicine, University of Utah, Salt Lake City, UT 84132, USA; 3 Departments of Epidemiology and Statistics, Fielding School of Public Health, University of California, Los Angeles, CA 90095, USA; cochran@ucla.edu; 4 UCLA Center for Bridging Research Innovation, Training and Education for Minority Health Disparities Solutions (BRITE), Los Angeles, CA 90095, USA; mays@ucla.edu; 5 Program for Research on Black Americans, Institute of Social Research, Ann Arbor, MI 48106, USA; jraffrty@umich.edu (J.R.); rjtaylor@umich.edu (R.J.T.); 6 School of Social Work, University of Michigan, Ann Arbor, MI 48109, USA; 7 Departments of Psychology and Health Policy and Management, Fielding School of Public Health, University of California, Los Angeles, CA 90095, USA

**Keywords:** mental health, DSM-IV, race/ethnicity, nativity, psychopathology, Black Lives Matter

## Abstract

There is growing diversity within the Black population in the U.S., but limited understanding of ethnic and nativity differences in the mental health treatment needs of Black women. This study examined differences in the prevalence of psychiatric disorders, their persistence, and unmet treatment needs among Black women in the U.S. Data were from the National Survey of American Life, a nationally representative survey that assessed lifetime and twelve-month mood, anxiety, and substance use disorders according to the Diagnostic and Statistical Manual of Mental Disorders 4th Edition (DSM-IV) criteria, and mental health service use among those meeting disorder criteria. One in three African American women met criteria for a lifetime disorder, compared to one in three Caribbean women born within the U.S. and one in five Caribbean women born outside the U.S. About half of African American women with a lifetime disorder had a persistent psychiatric disorder, compared to two in five Caribbean women born within the U.S. and two in three Caribbean women born outside the U.S. African Americans had more persisting dysthymia and panic disorder and less persisting social phobia compared to foreign-born Caribbean women. Of the three groups, Caribbean women born within the U.S. were most likely to seek mental health treatment during their lifetime. These results demonstrate, despite a lower prevalence of psychiatric disorders in Black women, that there is a great likelihood their disorders will be marked by persistence and underscores the need for culturally specific treatment approaches. As Black immigrants in the United States are increasing in number, adequate mental health services are needed.

## 1. Introduction

Psychiatric epidemiologic surveys have estimated patterns of mental health and substance use disorders for adults in the United States (U.S.), including African Americans, since the 1980s, but the scope of mental health treatment needs of Black women varying in ethnicity and nativity is not fully known [1]. The first studies to characterize the prevalence of mental health disorders among women using population-based designs found Black women to have a similar or lower prevalence of disorders compared to White women, with some exceptions based on the type of disorder and subpopulation characteristics [2,3]. In the Epidemiologic Catchment Area (ECA) study in the 1980s, it was observed that African Americans had an equal or lower likelihood of major depressive disorder (MDD) compared to Non-Hispanic Whites. However, young African American women (age 18–24) in the ECA had higher rates of MDD than did young Non-Hispanic White women [4]. In the National Comorbidity Survey (NCS) in the 1990s, Black women had lower rates of MDD compared to White or Hispanic women, except for a finding of elevated prevalence of MDD for Black women ages 35–44 [5]. In the ECA, Black women were found to have a higher prevalence of drug abuse compared to White women, but there were no Black–White differences in alcohol abuse [6]. While later studies from the National Epidemiologic Survey of Alcohol and Related Conditions (NESARC), conducted in the early 2000s, found the prevalence of substance use disorders among Black women to also be similar to or lower than the prevalence observed in White women [7,8]. Other mood disorders (i.e., dysthymia, bipolar I/II) and common anxiety disorders were not reported for Black women in the ECA, NCS, or NESARC.

A limitation of the population-based studies of Black women’s mental health conducted prior to 2000 is that these studies primarily focused on Black vs. White differences, as samples were not drawn with attention to the variation within the Black population. Approximately twenty percent of the recent growth of the Black population in the U.S. is due to immigration [9], and Caribbean immigrants make up a large portion of the foreign-born Black population [10]. By 2000, the Caribbean Black population was larger and growing faster than other racial or ethnic populations, such as Cubans or Koreans [10]. In 2016, almost half of the 1.6 million Black immigrants in the U.S. came from the Caribbean countries of Jamaica and Haiti [11]. Caribbean Blacks and African-Americans have different cultural and historical colonial experiences that may portend different patterns as protective or predictive of mental health disorders [12].

Consistent with the healthy immigrant hypothesis, prior studies comparing the prevalence of mental health and substance use disorders based on nativity found foreign-born adults to have a lower prevalence of disorders than their U.S.-born counterparts [13,14,15,16], although many of these studies were conducted in Latino and Asian populations. Studies in the United Kingdom have generally found that the rate of mental disorders was comparable for Caribbean Black women and European White women [17,18]. It is not clear if results from the United Kingdom would generalize to the U.S. The National Survey of American Life (NSAL; 2001–2003), the first representative population-based psychiatric epidemiologic survey to sample African American and Caribbean populations in the U.S. [19], can help to answer this question for mood, anxiety, and substance use disorders (see Table 1).

For mood disorders, in addition to confirming the sometimes lower prevalence of MDD for African American versus White women [20,21,22,23], studies from the NSAL found no differences in the prevalence of MDD or dysthymia for African American and Caribbean Black women [12,22]; African American mothers had elevated rates of bipolar disorder [23]. When nativity was considered, U.S.-born Caribbean women were found to have higher rates of any mood disorder compared to foreign-born Caribbean women [24]. For anxiety disorders, the NSAL revealed that African American women have a higher lifetime prevalence of post-traumatic stress disorder (PTSD) compared to Caribbean Black women [12]; there were no ethnic differences in the prevalence of other measured anxiety disorders, such as generalized anxiety disorder or panic disorder [12,22,25]. Foreign-born Caribbean women also had lower rates of any anxiety disorder compared U.S.-born Caribbean Black women. For substance use disorders, African American women were found to have a higher prevalence of alcohol abuse compared to Caribbean Black women [12]. Moreover, when nativity was considered, foreign-born Caribbean women were found to have an even lower prevalence of any substance use disorder compared to U.S.-born Caribbean or African American women [12,24,26]. However, research into the prevalence of specific mental health or substance use disorders in foreign-born Caribbean women, such as MDD or panic disorder, remains sparse [27].

**Table 1:** Findings from previous population-based studies of specific DSM-IV mood, anxiety, and substance use psychiatric disorders in African American and Caribbean women in the U.S.

Psychiatric Disorder	Population-Based Study	Diagnostic Criteria	Prevalence Observation Period [Reference no.] ^a^	Rates of Disorder by Ethnicity?	Rates of Disorder by Nativity?	Groups of Women Compared	Main Finding
**Mood Disorders**							
Major Depressive Disorder	ECA	DSM-III	6 mo. [28]	No	No	Black < White	Rates of MDD among Black women are less than or equal to that of White women.
	ECA	DSM-III	6 mo. [4], 12 mo., [29]	No	No	Black ≤ White	
			Lifetime [4]	No	No	Black ≤ White	(same as above)
	NCS	DSM-III R	30 day [5]	No	No	Black ≤ White	(same as above)
	NSAL	DSM-IV	12 mo., Lifetime [22,23]	Yes	No	NHW > CB = AA	(same as above)
	NSAL	DSM-IV	12 mo. [20], Lifetime [20,21]	No	No	AA ≤ White	(same as above)
	NSAL	DSM-IV	Lifetime [12]	Yes	No	AA = CB	No ethnic differences observed among women.
	NSAL	DSM-IV	12 mo., Lifetime [30,31]	No	No	AA only	Sociodemographic correlates of MDD differ for AA men compared to AA women.
Dysthymia	NSAL	DSM-IV	12 mo. Lifetime [23]	Yes	No	AA = CB = NHW	No ethnic differences observed among women.
	NSAL	DSM-IV	Lifetime [12]	Yes	No	AA = CB	No ethnic differences observed among women.
Bipolar Disorder I, II	NSAL	DSM-IV	12 mo., Lifetime [23]	Yes	No	AA ≥ CB = NHW	Rates of bipolar disorder are sometimes higher for AA women.
	NSAL	DSM-IV	Lifetime [12]	Yes	No	AA = CB	No ethnic differences observed among women.
**Any Mood Disorder**	NSAL	DSM-IV	12 mo. [24], Lifetime [12,24,32]	Yes	Yes	AA = CB; U.S.-born CB > Foreign-born CB	No ethnic differences observed among women. Foreign-born status appears protective for women.
**Anxiety Disorders**							
Agoraphobia	NSAL	DSM-IV	12 mo. [25], Lifetime [12]	Yes	No	CB = AA = NHW	No ethnic difference observed among women.
Post-traumatic Stress Disorder	NESARC	DSM-IV TR	Lifetime [33]	No	No	NHB = NHW	No ethnic differences observed among women.
	NSAL	DSM-IV	12 mo. [25]	Yes	No	CB = AA = NHW	No ethnic differences observed among women.
	NSAL	DSM-IV	Lifetime [12]	Yes	No	AA > CB	AA women have higher prevalence of PTSD.
Social Phobia	NSAL	DSM-IV	12 mo. [25]	Yes	No	NHW > CB = AA	White women have highest rates of social phobia
General Anxiety Disorder	NSAL	DSM-IV	12 mo. [25], Lifetime [12]	Yes	No	NHW > CB = AA	White women have the highest rates of GAD.
Panic Disorder	NSAL	DSM-IV	12 mo. [25], Lifetime [12]	Yes	No	CB = AA = NHW	No ethnic differences observed among women.
**Any Anxiety Disorder**	NSAL	DSM-IV	12 mo. [24], Lifetime [12,24,32]	Yes	Yes	AA > CB; U.S.-born CB > Foreign-born CB	AA women have higher rates of anxiety disorders compared to CB women. Foreign-born status appears protective for women.
**Substance Use Disorders**							
Alcohol Abuse	ECA ^b^	DSM-III	6 mo. [6,28]	No	No	Black = White	There are few racial differences in the prevalence of alcohol disorders among Black and White women.
	NESARC	DSM-IV	12 mo. [7]	No	No	Black < White	
	NSAL	DSM-IV	Lifetime [12]	Yes	No	AA > CB	AA women have higher rates of alcohol abuse
Alcohol Dependence	NESARC	DSM-IV	12 mo., Lifetime [7,8]	No	No	Black = White	No racial differences among women.
	NSAL	DSM-IV	Lifetime [12]	Yes	No	AA = CB	No ethnic differences among women.
Drug Abuse	ECA ^c^	DSM-III	6 mo. [28]	No	No	Black > White	Rates of drug use are sometimes higher for black women than for white women
	NSAL	DSM-IV	Lifetime [12]	Yes	No	AA = CB	No ethnic differences among women.
Drug Dependence	ECA^c^	DSM-III	6 mo. [28]	No	No	Black = White	No racial differences among women.
	NSAL	DSM-IV	Lifetime [12]	Yes	No	AA = CB	No ethnic differences among women.
**Any Substance Use Disorder**	NSAL	DSM-IV	12 mo. [24,26], Lifetime [12,24,26,32]	Yes	Yes	AA > CB; U.S.-born CB > Foreign-born CB	AA women have higher rates of substance disorders compared to CB women. Foreign-born status appears protective for women.
**Any of the Above Disorders**	NSAL	DSM-IV	12 mo. [24], Lifetime [12,24]	Yes	Yes	AA > CB; U.S.-born CB > Foreign-born CB	Rates of having at least one measured disorder are higher for AA women compared to CB women. Foreign-born status appears protective.

Abbreviations. ECA = Epidemiologic Catchment Area Study (1980–1983); NCS = National Comorbidity Survey (1990–1992); NCS-R = National Comorbidity Survey Replication (2001–2003); NSAL = National Survey of American Life (2001–2003); NESARC = National Epidemiologic Study of Alcohol and Related Conditions, Wave I (2001–2002); AA = African American; CB = Caribbean Black; NHW = Non-Hispanic White; DSM-IV = Diagnostic and Statistical Manual of Mental Disorders, 4th Edition ^a^ Number in bracket refers to bibliographic reference; ^b^ Rates of alcohol or dependence; ^c^ Rates of drug abuse or dependence.

While the NSAL and earlier psychiatric epidemiologic studies provide insight into the prevalence of mental health and substance use disorders, prevalence alone does not capture the full story for Black women. Indeed, prior studies of the general population found that disorder prevalence is often lower in Black versus White populations, and when present these disorders are more severe, persisting in their course, and more often untreated [22,34]. In one study of Black men’s mental health from the NSAL, foreign-born Caribbean men were more likely than other Black men to have persisting anxiety disorders, such as generalized anxiety disorder and social phobia [35]. Thus, the sometimes-observed lower prevalence of disorders can mask the real burden of mental health disorders within the Black population.

To our knowledge, prior studies have not triangulated information on prevalence, persistence, and service utilization for Black women differing in ethnicity and place of birth. In addition, we rarely see mental health services attending to these differences as part of a goal to provide culturally specific mental health interventions. Black women, compared to other racial or ethnic groups, are less likely to be married and be head of their households [36]; this sets up a dynamic for children and other dependents to be impacted by mothers whose mental health needs are not being addressed. Research into the mental health of Black women is especially important at present when the Black Lives Matter movement has called attention into the ways in which systemic racism weighs on Black women in the U.S., from increased interactions with the justice system for themselves and others in their households [37] to disparities in maternal mortality [38]. Understanding the history of the psychiatric disorder patterns experienced by diverse groups of Black women could help with developing culturally tailored solutions to improve women’s mental health currently and provide cause for acceleration in addressing inequities in the field of mental health treatments going forward.

## 2. Study Aim

Drawing on data from the NSAL, the goal of this study is to determine the prevalence, persistence, and unmet treatment needs of mood, anxiety, and substance use disorders among African American and Caribbean women in the U.S. We further examine the profile of mental health and substance use disorders for U.S.-born and foreign-born Caribbean women to better understand the diversity of mental health issues in Black women in the U.S. Our goal is to provide insights into how the patterns of various mental health and substance use disorders may change relative to nativity, the persistence of the disorders, and the unmet treatment needs of Black subpopulations of diverse women.

## 3. Methods

### 3.1. Procedures

This cross-sectional study uses data from the NSAL (2001–2003), an epidemiologic survey of psychiatric disorders aligned with the Diagnostic and Statistical Manual of Mental Disorders 4th Edition [DSM-IV] criteria, and mental health service utilization among Black Americans [39]. As described elsewhere, the NSAL is the largest survey of psychiatric disorders of Black Americans to date, as well as the first nationally representative survey of Black Caribbeans [40]. The four-stage probability sampling design focused on U.S. households where at least one Black adult (age 18 or older) resided in the contiguous U.S. To ensure adequate representation of Black adults of Caribbean descent, the NSAL oversampled geographic areas with a high density (at least 10%) of Black Americans who were also of Caribbean heritage.

Trained interviewers administered surveys from January 2001 to March 2003. All participants provided written informed consent and were compensated for their time. The NSAL response rates ranged from 70.7% among African Americans to 77.7% among Caribbean Blacks. Population-based sampling weights were calculated to minimize potential bias related to unequal probability of selection and participant non-response; the weights included a post-stratification correction to allow researchers to provide generalizable estimates of psychiatric disorder prevalence for Black Americans [41].

### 3.2. Ethics

The NSAL was conducted in accordance with the Declaration of Helsinki and the Institutional Review Board at the University of Michigan approved the study protocol (IRB: B03-00004038-R1). The NSAL data are publicly available through the Interuniversity Consortium for Political and Social Research (ICPSR): https://www.icpsr.umich.edu/web/ICPSR/studies/20240/datadocumentation.

### 3.3. Participants

Of the 6072 participants in the NSAL who completed surveys, 2299 women were African American and 978 women were of Caribbean descent. In the present study, we excluded data from 31 African American women born outside of the U.S. because the small sample size could lead to privacy and confidentiality concerns, as well as unstable estimates. We also excluded data from 26 African American women and 9 Caribbean Black women due to missing nativity status. The final analytic sample for this study included 2242 U.S.-born African American women, 264 U.S.-born Caribbean Black women, and 705 foreign-born Caribbean Black women.

### 3.4. Measures

#### 3.4.1. Ethnicity and Nativity

Participants self-reported their race, ancestry or ethnicity, and country of birth. Utilizing rules for racial or ethnic classification from the U.S. Census, we created three groups: U.S.-born African American, U.S.-born Caribbean, and foreign-born Caribbean women. This allowed us to examine not only the role of race in mental health prevalence, persistence, and unmet needs, but also the roles that ethnicity and nativity might contribute to these clinical and public health concerns. U.S-born Caribbeans included women who identified as Black, reported ancestral ties to a Caribbean country, and reported being born within the U.S. The foreign-born group included Caribbean Black women who reported being born in a country other than the U.S.

#### 3.4.2. Psychiatric Disorder Prevalence

The presence of probable lifetime and twelve-month psychiatric disorders was assessed using the World Mental Health version of the World Health Organization’s Composite International Diagnostic Interview (WHO-CIDI) [19,42]. The WHO-CIDI is a fully structured interview used to assess the prevalence of DSM-IV psychiatric disorders. In this study, the focus was the examination of three mood disorders (i.e., major depressive disorder, dysthymia, bipolar disorders I and II), five of the DSM-IV anxiety disorders (agoraphobia, PTSD, social phobia, general anxiety disorder, panic disorder), and four of the substance use disorders (alcohol abuse, alcohol dependence, drug abuse, and drug dependence). We also estimated the lifetime and twelve-month prevalence for panic attacks.

#### 3.4.3. Persistence of Disorders

We classified each psychiatric disorder as being persistent or not. As in other studies, persistence was defined as the proportion of adults with a lifetime psychiatric disorder who continued to meet diagnosis criteria in the twelve months that proceeded their interview date [34,43]. Our persistence analysis focused on the subsample of 874 African American and Caribbean Black women who (1) met criteria for a lifetime psychiatric disorder and (2) reported an age of onset at least two years prior to the date of their interview [35]. This measure is a proxy for identifying those with a disorder lasting more than twelve months [22,34]. The examination of persistence could illustrate how race combined with other statuses may be a factor in the experience of psychiatric disorders.

#### 3.4.4. Mental Health Service Utilization

Participants in the study were coded as having a lifetime of mental health service utilization if they selected any response that indicated that they had sought help for nerves, emotions, mental health, or use of alcohol or drugs from a medical provider (i.e., general practitioners), medical specialist (e.g., cardiologist), medical professionals (e.g., nurse), or mental health provider (i.e., psychologist, psychiatrist, counselor, or social worker in a mental health setting). We coded participants as positive for lifetime mental health service utilization if they affirmed seeking help from a medical or mental health provider at any time during their lifetime. Similarly, we coded participants as positive for twelve-month service utilization if they sought help from a medical or mental health provider in the twelve months prior to the interview.

### 3.5. Statistical Analysis

We conducted analyses in four steps. In the first step, we used design-adjusted cross-tabulations to determine the lifetime and prior twelve-month prevalence estimates of each of the measured mood, anxiety, and substance use disorders; the persistence of those disorders; and mental health service use across the three subgroups of Black women. As in prior work, the percentages here are weighted study proportions and the standard errors account for the complex survey design [35].

In the second step, we used multivariable logistic regressions to estimate differences in the prevalence of psychiatric disorders (lifetime and twelve-month disorders) among women varying in U.S. nativity and Caribbean heritage. In these analyses, we ran one set of models to compare the adjusted odds of meeting the DSM-IV disorder criteria for the Black Caribbean groups compared to African American women. Then, we ran another set of models to evaluate nativity differences in the adjusted odds of meeting the DSM-IV criteria within the Caribbean Black sample (i.e., the African Americans were excluded). The prevalence analyses, run separately for the lifetime and twelve-month disorders, controlled for sociodemographic characteristics shown in prior research to correlate with risk for a psychiatric disorder [43]: age, household income, poverty status (a ratio of family income to the U.S. census poverty threshold in 2001), education, employment status, marital status, and geographic region of the U.S.

In the third step, we used multivariable logistic regression analyses to test for differences in the persistence of each measured disorder among women varying in U.S. nativity and Caribbean heritage. As described above, the sample was restricted to participants whose disorder began at least two years prior to the interview. The first set of models compared the odds of meeting the twelve-month criteria for a disorder among the Black Caribbean groups compared to U.S.-born African American women. In the second set of models that excluded African Americans, we evaluated nativity differences among Caribbean Black women. The persistence analyses controlled for participant characteristics thought to contribute to a more chronic or persisting course of disorder [44,45,46,47,48,49]: age (18–30, 31–50, 51+), education (high school or less vs. other), marital status (married vs. other), poverty status (household income below 100% federal poverty level vs. other), and age of disorder onset [34,35].

In the fourth step, we used multivariable logistic regressions to test for group differences in the likelihood of mental health service use among those who met lifetime and twelve-month criteria for each mood, anxiety, and substance use disorder. As with the prevalence and persistence analyses above, we first ran the models to test for ethnicity-related differences in the odds of seeking mental health services, and then ran the models to test for nativity-related differences. The models of mental health service use each controlled for predisposing, enabling, and need factors, which are commonly associated with patients’ access to and use of mental health services [50,51,52]: age (18–30, 31–50, 51+), education (high school or less vs. other), marital status (married vs. other), poverty status (household income below 100% federal poverty level vs. other), and insurance status at the time of the interview.

All statistical analyses were conducted in SAS (9.1, SAS Institute Inc., Cary, NC, USA), included survey weights to provide population-generalizable estimates, and estimated variances according to the complex sampling design of the NSAL [53]. The survey weights were calculated to account for the unequal probability of selection and survey response and for post-stratification. The standard errors for estimates in the Black Caribbean groups were often larger than the standard errors for estimates among African American women because the Caribbean Black sample was smaller and more clustered than the African American sample.

## 4. Results

Characteristics of the sample are presented in Table 2. Compared to African American women, U.S.-born Caribbean Black women were younger and reported higher average household incomes. Additionally, U.S.-born Caribbean Black women, as compared to African American women, tended to report higher levels of education and were less likely to live in households where income was below the federal poverty level. Consistent with U.S. residential patterns, a greater proportion of African American women resided in the South compared to both groups of Caribbean Black women. The women did not differ significantly in their current employment, marital, or insurance status.

### 4.1. Lifetime Prevalence of Mood, Anxiety, and Substance Use Psychiatric Disorders

Approximately one-third of the U.S.-born African American and Caribbean women met criteria for a lifetime mood, anxiety, or substance use disorder, while only one in five foreign-born Caribbean women met such criteria (see Table 3, below). Approximately half of Black women born in the U.S with a lifetime disorder also met the criteria for a second lifetime disorder.

The patterns of differences in the prevalence of any lifetime disorder were largely mirrored when mood, anxiety, and substance use disorders were considered separately. In the overall sample, 15% of Black women met criteria for a lifetime mood disorder, 25% for a lifetime anxiety disorder, and 6% for a lifetime substance use disorder. With only one exception (i.e., dysthymia), there were no statistically significant differences in the rate of lifetime disorders between African American women and Caribbean women born in the U.S. However, foreign-born Caribbean women, compared to African Americans or U.S.-born Caribbeans, had a lower lifetime prevalence of major depressive disorder, agoraphobia, PTSD, panic attack, and each of the measured substance use disorders. Only 1% of foreign-born Caribbean women met the criteria for any lifetime substance use disorder.

### 4.2. Twelve-Month Prevalence of Mood, Anxiety, and Substance Use Psychiatric Disorders

Approximately one out of five African American women and one out of seven Caribbean women in this population-representative study met criteria for a psychiatric disorder in the twelve months prior to the interview (Table 3). Of those, about half of African American women and one-quarter of foreign-born Caribbean women met the criteria for two or more disorders in the preceding twelve months. There were no statistical differences between groups in the prevalence of having at least one psychiatric disorder in the past twelve months. However, the prevalence of having two or more psychiatric disorders in the preceding twelve months was higher in African American women compared to foreign-born Caribbean women.

For Black women in general, the prevalence of meeting the criteria for a psychiatric disorder in the prior 12 months was 8% for a mood disorder, 14% for an anxiety disorder, and 2% for a substance use disorder. There were some ethnic variations in the prevalence of specific mood, anxiety, and substance use disorders in the past 12 months. Compared to African American women, U.S.-born Caribbean women were less likely to meet criteria for dysthymia or alcohol dependence, but more likely to meet the criteria for panic attacks. In addition, African American women had a higher prevalence compared to foreign-born Caribbean women of dysthymia, panic disorder, any anxiety disorder, alcohol abuse, alcohol dependence, drug dependence, and any substance use disorder.

Within the Caribbean samples, foreign-born Caribbean women were less likely than U.S.-born Caribbean women to meet recent criteria for panic attacks, alcohol abuse or dependence, and drug abuse. We were unable to obtain adjusted group differences in the prevalence of bipolar I/II or drug dependence between the U.S. and foreign-born Caribbean Black women due to the very low prevalence of these disorders in each of the Caribbean groups.

### 4.3. Persistence of Mood, Anxiety, and Substance Use Psychiatric Disorders in Black Women in the U.S.

Over half of the women who met criteria for a lifetime psychiatric disorder were also classified as having a persistent disorder (Table 4). The percentage of disorders classified as being persistent was highest for anxiety (57.4%) and mood disorders (51.5%) and lowest for lifetime substance use disorders (29.7%). Among the three groups, foreign-born Caribbean women had the highest percentage of persistent disorders. They were more likely than U.S.-born Caribbean women to still meet DSM-IV criteria for any disorder in the past twelve months.

The patterns of ethnic and nativity differences in psychiatric persistence varied somewhat by disorder. African American women were more likely than both groups of Caribbean women to continue to meet DSM-IV criteria in the twelve months prior to the interview for dysthymia. African American women were less likely than foreign-born Caribbean women to continue to meet criteria for bipolar I/II or social phobia in the prior twelve months; but more likely than foreign-born Caribbean women to meet persistence criteria for panic disorder.

Within the Caribbean sample, foreign-born women were less likely than U.S.-born Caribbean women to meet criteria for panic attack and drug dependence in the past year; they were more likely to still meet criteria for any anxiety disorder, alcohol abuse, and alcohol dependence. We were unable to estimate nativity differences in the adjusted odds of persisting bipolar disorder I/II due to sparse numbers of Caribbean women meeting criteria in the past 12 months.

### 4.4. Lifetime Utilization of Mental Health Services

Two-thirds (66%) of Black women who met criteria for at least one of the measured psychiatric disorders also indicated that they had sought help from a general medical provider or a specialty mental health provider at some point during their lifetime. Among the entire sample, 72% of those with a lifetime mood disorder reported having used mental health services, which was similar to the portion of Black women with an anxiety disorder (68%) or a substance use disorder (69%).

Comparisons of mental health service use among the three groups of Black women revealed several significant differences (Table 5). Among women with any lifetime disorder, U.S.-born Caribbean women had the highest prevalence of lifetime mental health service use. This pattern was also observed for the subsample of women with two or more lifetime disorders, as well as for those with any mood, anxiety, or substance use disorder. In addition, U.S.-born Caribbean women had higher rates of lifetime mental health service use compared to African American women with diagnoses of major depressive disorder, bipolar I/II, social phobia, generalized anxiety disorder, and each of the substance use disorders.

Within the Caribbean sample, rates of mental health service use were much higher for U.S.-born Caribbean women compared to foreign-born Caribbean women. This pattern was observed for those with major depressive disorder, bipolar disorder I/II, any mood disorder, agoraphobia, social phobia, any anxiety disorder, drug abuse, drug dependence, and any substance use disorder.

### 4.5. Past-Year Utilization of Mental Health Services

Only one in four Black women (28%) who met criteria for a psychiatric disorder in the twelve months prior to the survey also reported seeking mental health care services in the twelve months prior to the interview. Among Black women as a group, 38% of those who met criteria for a mood disorder, 25% of those with an anxiety disorder, and 35% of those with a substance use disorder received services in the year prior to the interview.

There were only a few ethnic differences in mental health service use in the past twelve months (Table 5). Specifically, African American women with a past twelve-month disorder of bipolar I/II, panic disorder, or alcohol abuse had lower rates of recent mental health service use compared to U.S.-born Caribbean women with the same disorders. Compared to African American women, rates of mental health service use in the past twelve months were lower for foreign-born Caribbean women with major depressive disorder, any mood disorder, social phobia, or panic attack; and higher for foreign-born Caribbean women with bipolar disorder I/II, agoraphobia, alcohol abuse, or alcohol dependence.

In the Caribbean cohort, foreign-born women with any recent disorder were less likely than U.S.-born Caribbean women to have reported using mental health services in the past year. These patterns of nativity differences in service utilization were also observed for women with dysthymia, social phobia, panic attack, or any anxiety disorder. However, the patterns of nativity differences in mental health service use were reversed for women with substance use disorders; foreign-born Caribbean women who met criteria for drug abuse or any substance use disorder in the past twelve months had higher rates of recent mental health service use compared to U.S.-born Caribbean women with the same diagnoses.

Nativity differences in the adjusted odds of using mental health services could not be estimated for women with agoraphobia, alcohol abuse, alcohol dependence, or drug dependence due to small numbers and limited variation in responses. For instance, in the very small samples of Caribbean women with recent bipolar disorder I/II or alcohol abuse, all U.S.-born women with these diagnoses reported using mental health services. None of the Caribbean women in our sample who met the criteria for drug dependence in the past twelve months reported using mental health services in the prior 12 months.

## 5. Discussion

This study sought to illuminate the diverse mental health needs of Black women in the U.S. by combining information on the prevalence, persistence, and treatment utilization patterns of women varying in ethnic background and country of birth. An additional goal was to determine if Black women, similar to Black men, experienced substantial persistence of mental health and substance use disorders when prevalence criteria were met. Indeed our results do call attention to the fact that similar to Black men [35], one-half of Black women with a disorder suffer from persistence of that disorder. In the U.S., this is compounded by the fact that some Black women are not seeking treatment for these disorders [23], which puts them at risk for the consequences of untreated mental health.

### 5.1. Patterns of Differences in Mental Health Disorder Prevalence

Our findings build on prior literature by presenting estimates of lifetime and twelve-month rates of specific mood, anxiety, and substance use disorders for foreign-born Caribbean women to better aid mental health services planning for this growing population. Consistent with studies of mental health and substance use among African and Caribbean immigrants carried out in other countries [54,55,56,57], we found Black women who have migrated from the Caribbean had lower rates of psychiatric disorders than Black women born in the U.S., especially for substance use disorders. These results are consistent with the healthy immigrant literature, which finds that foreign-born populations often have better health and mental health outcomes [13,16,54,57,58,59,60,61]. In addition, this study contributes to the literature by demonstrating the chronic course of mental health and substance use disorders, such as dysthymia and panic disorder in African American women, which appear to persist even for women who have accessed mental health services during the lifetime.

### 5.2. Understanding Differences in Psychiatric Disorder Persistence

Our findings of differences in the persistence of disorders based on U.S. versus foreign-born nativity does not fully align with the healthy immigrant literature—a finding that may be less true for conditions of mental health. The rates of persistence were generally comparable for the foreign-born Caribbean women and U.S.-born African American women. It was the U.S.-born Caribbean women who, except for panic attacks and drug dependence, stood out as having particularly low rates of persistence. Foreign-born Caribbean women had the highest rate of persisting social phobia. We might expect persistence to be lower among U.S.-born Caribbean Black women if they are differentially exposed to stressors commonly associated with mental illness or if there are differences in the amount, quality, or access to mental health treatment. Another possible explanation is that Caribbean Black women differentially weather stressors. One NSAL study found more social stressors to be associated with higher rates of depressive symptoms for African American women than for Caribbean Black women [31]. In our study, the U.S.-born Caribbean women generally had more favorable socioeconomic standing in terms of their education and household income compared to African American women, which could be another contributing factor to the differences in persistence we see [62].

Two unique issues are raised by our findings of ethnic and nativity differences in persistence. First, it is assumed that the marginalized position of being Black in the U.S. will result in greater exposure to stressors often shown to be associated with worse mental health outcomes [63,64,65]. Yet there is something about the social patterning of race plus nativity that results in differences in mental health experience, as well as in seeking treatment. The advantage of household income and education may confer resources to better weather stresses and potentially results in fewer cumulative exposures. The U.S.-born Caribbean women may be unique from the other groups of Black women in their available resources or in the urgency or intensity of treatment when a mental health problem arises. Indeed, in the current study, U.S.-born Caribbean women had higher rates of lifetime mental health service use, even when compared to African American women. This raises a second point in need of further research, which is that differences in cultural socialization between being Black in the U.S. versus early socialization of mental health coping in the foreign-born Black woman. Additional research into mental health coping and other resources to weather stressors could inform the design of interventions to address unmet treatment needs within various groups of Black women in the U.S.

### 5.3. Implications for Mental Health Services

In our study, foreign-born Caribbean women had the lowest rates of treatment utilization, whether across the lifetime or within the twelve months prior to the interview. These findings parallel those for Latino and Asian immigrants [66,67]. Immigrants often face additional barriers to treatment due to residency status or concerns about bringing shame to their community [68]. A few studies have documented high rates of mental health stigma among Caribbean immigrants [69,70], which may contribute to their low rates of treatment seeking. Whether these barriers have a differential harmful impact on Caribbean women is unknown. Because untreated mental health disorders are so closely linked to cardiovascular disease and metabolic disorders [71,72,73,74], failure to engage Caribbean women in evidence-based mental health treatment is likely to have cascading consequences for not only their emotional health, but for their physical health as well.

There were more statistical differences in the rate of treatment across the lifetime than in the twelve months prior to the interview. Part of this may be due to the smaller sample size (and thus larger standard errors) that accompanied the twelve-month analyses. However, it could also be the case that there are differences in the time to treatment or in the length or quality of treatment, which may explain the different patterns of results. Unfortunately, we do not know, and this is an area for further study. A majority of the population-based studies involving Black Americans that have examined the time to treatment [75], the quality of treatment received [76], or treatment retention [22,77] have focused on Black vs. White differences and have not examined the experiences of Caribbean immigrants. One study from the NSAL found that Caribbean migrants had more favorable mental health service experiences compared to U.S.-born Caribbeans [78], but these findings were not reported specifically for Black women.

The results of our analyses indicate that there are cultural differences in several factors, such as the tendency to seek care, chronicity in different disorders, and prevalence of these disorders. Those primary care practices that reside in neighborhoods whose patient populations contain a number of Black women should ensure that they are collecting subgroup ethnicity and nativity data so that they can respond adequately to differences in mental health needs among Black women. It will not be adequate for clinics and health care systems to merely know that their populations are Black, but knowing patients’ ethnicity and nativity may be useful in anticipating and designing effective care for mental health and substance use problems.

### 5.4. Study Limitations

This study has four primary study limitations. First, the prevalence rates presented here only generalize to non-institutionalized Black women. African American women are more likely than White women to be represented in the prison system [37] and rates of psychiatric disorders are much higher among incarcerated women than community samples [79,80]. Therefore, our prevalence estimates from a household-based sample underestimate the burden of mental illness among Black women in the U.S. as well as their unmet need for treatment.

Second, there is evidence from the clinical reappraisal studies that the WHO-CIDI did not perform equally well in identifying psychiatric disorders across ethnic groups in the NSAL. The evidence suggests that the WHO-CIDI picked up a larger portion of false positive cases among the Caribbean population [24]. Therefore, caution should be exercised in thinking about the prevalence of disorders reported for Caribbean women as our findings may have overestimated their prevalence and needs.

Third, this study was limited by low statistical power to examine ethnic differences in persistence or mental health service utilization for some disorders (such as bipolar disorder or alcohol use disorder) that are less prevalent among Black women. We also were limited by low statistical power to further examine mental health differences within subgroups of Caribbean women (e.g., different immigration cohorts or countries of origin), or to estimate mental health disorder prevalence, persistence, and treatment utilization for foreign-born women emigrating from African countries. Previous studies have found that immigrants’ mental health varies by country of origin, the disorder being studied, age at immigration, and time in the U.S.

Finally, the use of data from 2001–2003 raises questions about the generalizability of findings to the present time. It is likely that the unmet mental health needs of African American and Black Caribbean women in the U.S. could be higher in 2020 than they were in 2003. For example, the 2008 economic recession disproportionately affected Black households in the U.S. and may have contributed to increased mental health disparities [81,82,83,84]. Most recently, the coronavirus disease (COVID-19) pandemic has led to higher death rates in both the elderly and younger groups in the Black population [85,86,87,88]. This is occurring without usual burial and grief relief rituals. The social isolation policies under COVID-19 are also contributing to reduced income, food insecurity, and deferred outpatient care, which are all factors in unmet mental health treatment needs [89,90,91,92,93,94,95]. In addition, the Black Lives Matter movement has highlighted the concerns of Black women about their own safety, as well as that of their male family members, in relation to police shootings.

Despite the age of the study data, there are still several compelling reasons why the NSAL offers relevant insights for use in current mental health service planning and clinical care. Currently, the NSAL remains the only data set with enough cases within a representative population sample of Black racial and ethnic minority adults to conduct meaningful analyses on psychiatric disorders. Before the advent of the NSAL data set, reliable, nationally representative information on psychiatric disorders for Black women was scarce or relied on data that could be only abstracted from treatment settings [1]. In light of the historic scarcity of national probability-based data sets on Black mental health, the rising numbers of suicides in Black Americans, and the worsening of factors that contribute to mental health, as highlighted by COVID-19 and Black Lives Matter, the NSAL remains the best and most comprehensive data for the study of representative mental health in the Black female population of the U.S., despite its age.

### 5.5. Implications for Future Research

We recommend launching a new population-based study to determine the prevalence, persistence, and treatment needs of psychiatric disorders in Black women in order to provide updated estimates on the mental health of Black women following historic events and to illuminate the mechanisms through which Black women weather economic and social stressors. Critical to extending our findings would be larger subpopulation samples and assessment of migration characteristics (e.g., age, generational status) to further investigate the treatment and health service needs of different subpopulations of Black women. This is particularly important as ours and other studies have indicated significant differences in help-seeking patterns, perceived need for treatment, and stigma concerns of Caribbean women compared to U.S.-born African American women [27,69]. Moreover, there has been a surge in African migration in recent years [11], and the mental health service needs of African immigrants remain poorly understood from a population-based perspective [1]. As demonstrated in our study, failure to not separate Black women in terms of ethnicity and place of birth would obscure the true rates of perceived need and pathways of seeking treatment for U.S.-born African American women and fail to help us to identify those social and cultural conditions of immigrants adjusting to life in the U.S. that may put them at risk for psychiatric disorders [96].

## 6. Conclusions

Despite these limitations, this study makes an important contribution to the field, as it is among the first to bring together a trifecta of mental health service issues together to identify the mental health status, persistence of disorders, and unmet treatment needs among a nationally representative sample of African American women and U.S.-born, and foreign-born Caribbean Black women in the U.S. While the rates of psychiatric disorders among Black women are lower than the national estimates of psychiatric disorders in the U.S. from the National Comorbidity Survey Replication [44], nonetheless for those who do have a disorder there is a public health concern. Our result illustrate there was wide variation by ethnicity in the prevalence, course, and unmet treatment needs of their psychiatric disorders. As the U.S. continues to be the number one choice for a growing number of African and Caribbean women to live and raise their families, researchers and policymakers must attend to differences in this diversity that will allow us to target prevention efforts and mental health service planning to the specific needs of these Black subpopulations in the U.S. Results of this study help to underscore that despite being racially the same, variations in ethnicity and nativity result in distinctive vulnerabilities and mental health treatment needs.

## Figures and Tables

**Table 2:** Sociodemographic Characteristics of Black Women in the National Survey of American Life, 2001–2003.

	African American		Caribbean Black				
Sociodemographic Characteristics	U.S.-Born (n = 2242)		U.S.-Born (n = 264)		Foreign-Born (n = 705)		
	Weighted % (S.E.)						X^2^
Age							
18–29	24.4	(1.2)	42.9	(4.9)	19.5	(2.7)	
30–44	34.5	(1.2)	32.7	(4.4)	41.2	(3.0)	
45–59	23.8	(1.0)	10.2	(1.9)	23.4	(3.5)	
60 or greater	17.3	(1.1)	14.2	(4.8)	15.9	(2.1)	25.837 ***
Income							
Less than $18,000	37.2	(1.5)	24.8	(4.1)	25.0	(3.1)	
$18,000–31,999	26.4	(1.2)	28.6	(3.5)	29.8	(3.7)	
$32,000–54,999	20.2	(0.9)	17.7	(4.3)	23.5	(3.7)	
$55,000 or greater	16.2	(1.3)	28.9	(6.8)	21.7	(3.8)	19.011 **
Employment Status							
Working	62.9	(1.4)	69.6	(4.6)	71.4	(4.4)	
Not Working	37.1	(1.4)	30.4	(4.6)	28.6	(4.4)	5.090
Marital Status							
Currently Married	35.0	(1.3)	34.3	(6.3)	43.7	(4.7)	
Previously Married	32.3	(1.0)	22.3	(5.5)	30.1	(4.8)	
Never Married	32.7	(1.5)	43.5	(4.0)	26.2	(2.3)	8.926
Education							
Less than High School	25.1	(1.8)	15.8	(5.3)	22.9	(2.9)	
High School	36.6	(1.3)	26.6	(5.3)	32.5	(2.3)	
Some College	24.7	(1.1)	38.1	(5.4)	24.3	(3.1)	
College	13.6	(1.2)	19.5	(3.4)	20.3	(2.5)	18.568 **
Poverty Status							
In Poverty	29.3	(1.4)	21.9	(4.2)	19.0	(2.5)	
Not in Poverty	70.7	(1.4)	78.1	(4.2)	81.0	(2.5)	11.851 **
Geographic Region							
South	55.6	(2.3)	24.2	(7.1)	27.8	(6.3)	
Non-South	44.4	(2.3)	75.8	(7.1)	72.2	(6.3)	27.340 ***
Insurance Status							
Insured	82.6	(1.3)	80.8	(6.0)	80.0	(3.3)	
Uninsured	17.4	(1.3)	19.2	(6.0)	20.0	(3.3)	0.548
Citizenship							
U.S. Citizen					63.4	(3.5)	
Non-U.S. Citizen					36.6	(3.5)	

Abbreviations: U.S. = United States; S.E. = standard error. Note: ** p < 0.01, *** p < 0.001.

**Table 3:** Weighted prevalence of lifetime DSM IV/WMH-CIDI mood, anxiety, and substance use psychiatric disorders among black women in the National Survey of American Life.

	Lifetime DSM-IV Disorders						Twelve-Month DSM-IV Disorder					
	African American		Caribbean Black				African American		Caribbean Black			
DSM-IV Disorder	U.S.-Born (n = 2242)		U.S.-Born (n = 264)		Foreign-Born (n = 705)		U.S.-Born (n = 2242)		U.S.-Born (n = 264)		Foreign-Born (n = 705)	
	Weighted % (S.E.)						Weighted % (S.E.)					
**Mood Disorders**												
Major Depressive Disorder	14.4	(0.8)	22.6	(5.7)	11.2	(2.0) ^b^	8.2	(0.5)	7.5	(1.8)	7.0	(2.1)
Dysthymia	4.3	(0.5)	1.9	(0.7) ^a^	2.7	(1.1)	3.2	(0.5)	0.7	(0.4) ^a^	0.4	(0.2) ^a^
Bipolar Disorder I, II ^c^	1.4	(0.3)	0.9	(0.8)	1.0	(0.6)	1.0	(0.3)	0.7	(0.8)	0.5	(0.2)
Any Mood Disorder	14.8	(0.8)	22.6	(5.7)	11.4	(2.0) ^b^	8.5	(0.5)	7.5	(1.8)	7.2	(2.1)
**Anxiety Disorders**												
Agoraphobia	2.4	(0.4)	4.7	(3.7)	1.8	(0.8) ^b^	1.5	(0.2)	0.7	(0.4)	1.1	(0.7)
Post-traumatic Stress Disorder	12.2	(0.8)	9.9	(2.0)	7.8	(1.5) ^a^	4.9	(0.5)	3.8	(1.2)	3.0	(1.1)
Social Phobia	8.3	(0.8)	5.4	(2.2)	5.1	(1.2)	5.5	(0.6)	3.6	(1.3)	4.6	(1.1)
General Anxiety Disorder	5.6	(0.6)	6.8	(2.4)	3.1	(1.1)	3.2	(0.5)	2.5	(1.6)	1.4	(0.7)
Panic Disorder	4.4	(0.5)	4.1	(1.2)	2.5	(1.0)	3.1	(0.4)	2.4	(0.9)	0.8	(0.3) ^a^
Panic Attack	24.7	(1.0)	29.6	(3.9)	17.5	(3.3) ^a,b^	10.8	(0.8)	20.3	(4.8) ^a^	7.8	(2.0) ^b^
Any Anxiety Disorder	24.0	(1.1)	23.3	(4.1)	13.8	(1.7) ^a^	13.8	(0.9)	9.5	(2.5)	8.0	(1.4) ^a^
**Substance Use Disorders**												
Alcohol Abuse	5.2	(0.7)	4.6	(1.8)	0.6	(0.9) ^a,b^	1.2	(0.3)	0.4	(0.4)	0.2	(0.1) ^a,b^
Alcohol Dependence ^c^	2.5	(0.4)	2.6	(1.7)	0.2	(0.1) ^a,b^	0.9	(0.3)	0.0	(0.0) ^a^	0.1	(0.1) ^a,b^
Drug Abuse	3.3	(0.5)	4.5	(1.8)	0.6	(0.2) ^a,b^	1.0	(0.2)	1.2	(0.2)	0.1	(0.1) ^a,b^
Drug Dependence	1.3	(0.3)	2.3	(0.9)	0.4	(0.2) ^a,b^	0.3	(0.1)	0.5	(0.5)	0.1	(0.1)
Any Substance Use Disorder	6.5	(0.7)	5.8	(1.8)	1.0	(0.3) ^a,b^	2.1	(0.4)	1.6	(0.4)	0.3	(0.1) ^a,b^
**Any of the Above Disorders**	32.4	(1.1)	33.8	(5.5)	19.5	(2.1) ^a,b^	18.6	(0.9)	14.1	(3.4)	12.8	(2.1)
**2 or More of the Above Disorders**	15.4	(0.8)	17.3	(3.6)	8.3	(1.3) ^a^	7.8	(0.7)	5.9	(1.4)	3.0	(0.8) ^a^

Abbreviations: DSM IV = Diagnostic and Statistical Manual of Mental Disorders, 4th edition; WMH-CIDI = World Mental Health version of the World Health Organization’s Composite International Diagnostic Interview; U.S. = United States; S.E. = standard error. Pairwise comparisons of ethnic and nativity differences in the prevalence of psychiatric disorders obtained from multivariable logistic regressions. ^a^ Adjusted odds of meeting disorder criteria, compared to African American women, was statistically significant at *p* < 0.05. ^b^ Adjusted odds of meeting disorder criteria, compared to U.S.-born Caribbean women, was statistically significant at *p* < 0.05. ^c^ Multivariate pairwise comparisons of ethnic or nativity differences in the 12-month prevalence of specific disorders were not conducted due to the small samples.

**Table 4:** Proportion of Black women with a lifetime DSM-IV mood, anxiety, and substance use psychiatric disorder who met the criteria for a psychiatric disorder in the past 12 Months.

		African American		Caribbean Black			
DSM-IV Disorder		U.S.-Born		U.S.-Born		Foreign-Born	
	N	Weighted % (S.E.)					
**Mood Disorders**							
Major Depressive Disorder	370	51.8	(2.9)	29.4	(9.2)	58.9	(12.2)
Dysthymia	105	73.1	(5.4)	37.6	(14.5) ^a^	14.0	(5.2) ^a^
Bipolar Disorder I, II ^c^	25	70.5	(9.6)	--		100	(0.0) ^a^
Any Mood Disorder	379	52.2	(2.9)	29.4	(9.5)	58.8	(12.0)
**Anxiety Disorders**							
Agoraphobia	75	59.0	(6.6)	13.9	(13.2)	60.2	(16.6)
Post-traumatic Stress Disorder	287	40.6	(3.3)	38.0	(11.2)	34.5	(10.8)
Social Phobia	223	64.7	(4.0)	64.5	(9.0)	90.1	(5.1) ^a^
General Anxiety Disorder	141	56.1	(5.1)	34.4	(22.8)	44.5	(18.8)
Panic Disorder	124	70.4	(4.7)	54.4	(21.4)	27.2	(11.1) ^a^
Panic Attack	633	41.3	(2.7)	57.0	(12.4)	36.0	(13.2) ^b^
Any Anxiety Disorder	630	57.8	(3.0)	40.3	(10.1)	57.7	(6.3) ^b^
**Substance Use Disorders**							
Alcohol Abuse	132	23.1	(4.2)	8.3	(7.9)	19.9	(10.4) ^b^
Alcohol Dependence	58	36.4	(5.7)	--		25.8	(32.5) ^b^
Drug Abuse	90	23.7	(4.8)	26.8	(4.6)	10.6	(12.2)
Drug Dependence	40	23.7	(7.8)	22.9	(18.0)	14.8	(14.2) ^b^
Any Substance Use Disorder	168	29.8	(4.2)	27.7	(9.4)	25.0	(12.3)
**Any of the Above Disorders**	874	56.1	(2.3)	40.5	(10.7)	64.2	(6.3) ^b^

Abbreviations: DSM IV = Diagnostic and Statistical Manual of Mental Disorders, 4th edition; U.S. = United States; S.E. = standard error. Pairwise comparisons of ethnic and nativity differences in the persistence of psychiatric disorders obtained from multivariable logistic regressions. Analyses were restricted to those that first met the DSM-IV criteria for a lifetime disorder at least two years prior to the interview. ^a^ Adjusted odds of meeting persistence criteria, compared to African American women, was statistically significant at *p* < 0.05. ^b^ Adjusted odds of meeting persistence criteria, compared to U.S.-born Caribbean women, was statistically significant at *p* < 0.05. ^c^ Multivariate pairwise comparisons of nativity differences in the persistence of specific disorders were not conducted due to the small samples.

**Table 5:** Weighted percentages of African American vs. U.S.-born and foreign-born Caribbean Black women with a history of a DSM-IV mood, anxiety, or substance use psychiatric disorder that sought mental health services.

	Lifetime Service Use							Twelve-Month Service Use						
		U.S.-Born African American		Caribbean Black					U.S.-Born African American		Caribbean Black			
DSM-IV Disorder				U.S.-Born		Foreign-Born					U.S.-Born		Foreign-Born	
	N	Weighted % (S.E.)						N	Weighted % (S.E.)					
**Mood Disorders**														
Major Depressive Disorder	426	72.4	(2.3)	87.0	(5.3) ^a^	42.2	(11.3) ^a,b^	235	38.9	(3.7)	49.1	(16.3)	13.3	(6.5) ^a^
Dysthymia	108	82.6	(4.3)	56.1	(14.4)	85.3	(5.2)	71	39.5	(7.0)	26.1	(17.6)	11.5	(11.9) ^b^
Bipolar Disorder I, II ^c^	47	90.3	(3.9)	100	(0.0) ^a^	88.7	(6.1) ^b^	32	43.9	(9.7)	100	(0.0) ^a^	67.1	(0.0) ^a^
Any Mood Disorder	441	72.6	(2.3)	87.0	(5.6) ^a^	41.4	(11.0) ^a,b^	246	38.6	(3.6)	49.1	(17.3)	13.1	(6.4) ^a^
**Anxiety Disorders**														
Agoraphobia ^c^	77	60.2	(5.8)	90.4	(10.1)	10.6	(7.8) ^a,b^	46	6.7	(4.1)	--		12.3	(10.9) ^a^
Post-traumatic Stress Disorder	331	73.9	(3.2)	87.1	(6.5)	58.6	(10.0)	134	26.0	(5.4)	33.7	(19.5)	12.4	(6.7)
Social Phobia	228	64.0	(3.5)	88.9	(8.1) ^a^	43.8	(15.6) ^b^	158	22.6	(3.4)	56.5	(19.9)	5.2	(3.2) ^a,b^
General Anxiety Disorder	148	73.8	(4.6)	98.5	(1.6) ^a^	69.5	(18.4)	80	34.3	(6.4)	11.5	(10.8)	18.9	(11.8)
Panic Disorder	131	82.5	(4.4)	90.6	(9.1)	90.9	(6.3) ^a^	92	32.1	(7.0)	51.1	(12.0) ^a^	20.1	(11.9)
Panic Attack	715	62.0	(3.0)	64.9	(10.4)	45.4	(13.8)	346	30.9	(3.1)	47.8	(15.8)	10.7	(5.3) ^a,b^
Any Anxiety Disorder	662	67.9	(2.5)	89.9	(4.2) ^a^	58.4	(8.1) ^b^	383	25.3	(2.4)	33.9	(11.1)	12.6	(4.4) ^b^
**Substance Use Disorders**														
Alcohol Abuse ^c^	140	65.3	(4.9)	95.9	(2.1) ^a^	72.7	(13.6)	35	13.5	(6.5)	100	(0.0) ^a^	54.8	(0.0) ^a^
Alcohol Dependence ^c^	59	75.7	(6.0)	100	(0.0) ^a^	100	(0.0) ^a^	19	33.0	(10.8)	--		100	(0.0) ^a^
Drug Abuse	97	71.7	(5.4)	98.1	(2.0) ^a^	71.9	(10.7) ^b^	27	46.5	(10.6)	15.0	(6.4)	50.7	(25.0) ^b^
Drug Dependence ^c^	40	81.6	(3.9)	100	(0.0) ^a^	85.2	(14.2) ^a,b^	9	48.2	(8.4)	--		--	
Any Substance Use Disorder	175	67.9	(4.4)	96.8	(1.7) ^a^	68.4	(13.7) ^b^	56	34.5	(6.9)	35.3	(13.6)	44.2	(12.1) ^b^
**Any of the Above Disorders**	933	65.5	(1.9)	85.7	(4.5) _a_	47.0	(8.6) _b_	536	28.8	(2.3)	33.6	(10.3)	12.1	(4.2) ^a,b^
**2 or More of the Above Disorders**	432	75.6	(2.9)	92.7	(4.1) _a_	63.9	(8.4) _b_	209	33.1	(3.3)	49.9	(10.3)	20.4	(6.3)

Abbreviations: DSM IV = Diagnostic and Statistical Manual of Mental Disorders, 4th edition; U.S. = United States; S.E. = standard error. Pairwise comparisons of ethnic and nativity differences in the likelihood of seeking mental health services, obtained from multivariable logistic regressions. ^a^ Adjusted odds of mental health service use, compared to African American women, was statistically significant at *p* < 0.05. ^b^ Adjusted odds of mental health service use, compared to U.S.-born Caribbean women, was statistically significant at *p* < 0.05. ^c^ Multivariate pairwise comparisons of nativity differences in twelve-month service use could not be estimated due to small samples and lack of variation in service use. Note: -- indicates that weighted estimates could not be obtained because none of the lifetime cases reported using mental health services.
